# Seasonality of interactions between a plant virus and its host during persistent infection in a natural environment

**DOI:** 10.1038/s41396-019-0519-4

**Published:** 2019-10-30

**Authors:** Mie N. Honjo, Naoko Emura, Tetsuhiro Kawagoe, Jiro Sugisaka, Mari Kamitani, Atsushi J. Nagano, Hiroshi Kudoh

**Affiliations:** 1 Center for Ecological Research, Kyoto University, Otsu, 520-2113 Japan; 2 Faculty of Agriculture, Kagoshima University, Korimoto 1-21-24, Kagoshima, 890-0065 Japan; 3 Faculty of Agriculture, Ryukoku University, Otsu, 520-2194 Japan

**Keywords:** Plant ecology, Virus-host interactions, Microbial ecology

## Abstract

Persistent infection, wherein a pathogen is continually present in a host individual, is widespread in virus–host systems. However, little is known regarding how seasonal environments alter virus–host interaction during such metastability. We observed a lineage-to-lineage infection of the host plant *Arabidopsis halleri* with *Turnip mosaic virus* for 3 years without severe damage. Virus dynamics and virus–host interactions within hosts were highly season dependent. Virus accumulation in the newly formed leaves was temperature dependent and was suppressed during winter. Transcriptome analyses suggested that distinct defence mechanisms, i.e. salicylic acid (SA)-dependent resistance and RNA silencing, were predominant during spring and autumn, respectively. Transcriptomic difference between infected and uninfected plants other than defence genes appeared transiently only during autumn in upper leaves. However, the virus preserved in the lower leaves is transferred to the clonal offspring of the host plants during spring. In the linage-to-linage infection of the *A. halleri*–TuMV system, both host clonal reproduction and virus transmission into new clonal rosettes are secured during the winter–spring transition. How virus and host overwinter turned out to be critical for understanding a long-term virus–host interaction within hosts under temperate climates, and more generally, understanding seasonality provides new insight into ecology of plant viruses.

## Introduction

Plant viruses have been studied primarily as important pathogens of crops. They cause various diseases characterised by a combination of severe symptoms and high transmission rates [1–3]. However, in natural ecosystems, plant viruses have often been reported to spread widely in plant populations without causing critical damage to their hosts [4–6]. Under such circumstances, continued long-term presence of the virus in the host individuals (persistent infections) is expected in perennial plants [7–9]. For long-term persistence of both virus and host individuals, either the mechanism that keeps within-host virus accumulation low or the one that keeps virulence low even at high virus accumulation are required [10, 11].

In natural systems, within-host virus accumulation may largely change with seasons depending on virus replication and host growth. Regarding host populations, seasonality of infection rate and its determinant factors, such as vector activities and host susceptibility, have been studied [12–14]. Previous studies have also reported seasonality of symptoms [15, 16], suggesting the importance of seasonal dynamics on within-host virus accumulation as a determinant of virulence. However, little is known about the seasonal dynamics of within-host virus accumulation and host responses in natural plant populations.

The degree of virus accumulation in its host is likely to show seasonality because both viral replication and plant growth are highly temperature dependent. The optimal temperature for the replication of plant viruses varies ranging between 15 °C and 30 °C [17–20]. Similarly, the rate of leaf production is highly temperature dependent in plants [21–24]. In plants that remain green throughout the winter season, it has been reported that some species can produce leaves in low-temperature regimes between 5 °C and 10 °C [25]. The presence of a period with low within-host virus accumulation may restrict the cumulative effects of virus on host individuals during a year. Regarding the mechanisms to keep virus accumulation low, there are many studies on the antiviral defences of plants, such as RNA silencing, hypersensitive response, systemic acquired resistance, and their cross-talks [26–29]; however, whether the relative importance of these antiviral mechanisms varies across seasons is still unknown.

In this study, we investigated the seasonal dynamics of virus–host interactions using a perennial herbaceous plant, *Arabidopsis halleri* subsp. *gemmifera* (*A. halleri*, hereafter), and *Turnip mosaic virus* (TuMV), a virus infecting it in natural environments. In a natural population of *A. halleri* in central Japan [30], we found that TuMV infections were common but without severe symptoms [31, 32]. In our previous study, we applied RNA-Seq to simultaneously determine infection rate of all viruses and host responses in early summer, and found that 57% of examined plants were infected by TuMV, and a homologue of antiviral defence gene, *ARGONAUTE 2* (*AGO2*), was upregulated in TuMV-infected plants [31]. *Arabidopsis halleri* produces clonal rosettes, and we previously reported that TuMV was transmitted to 92% of clonal rosettes produced by TuMV-infected plants [32]. Due to the clonal rosette formation, *A. halleri* often forms plant patches that consist of multiple rosettes with a shared genotype. We first investigated whether a single lineage of TuMV persisted in the host plant patch for multiple years. We further examined seasonal patterns in TuMV distribution among leaves within a single plant, and experimentally tested whether those patterns were temperature dependent. Along with the seasonality of within-host virus accumulation, we evaluated the seasonality in host responses at the gene expression level by comparing host transcriptome (RNA-Seq) between infected and uninfected plants on representative dates during four seasons, i.e. spring equinox, summer solstice, autumn equinox, and winter solstice (hereafter referred to as SE, SS, AE, and WS, respectively).

## Materials and methods

### Study system

*Arabidopsis halleri* (L.) O’Kane & Al-Shehbaz subsp. *gemmifera* (Matsum.) O’Kane & Al-Shehbaz [Brassicaceae] is a perennial herb. The species flowers from April to May, and then, reproduce sexually by producing seeds and clonally by forming vegetative rosettes. Rosettes grow continually during summer and autumn, and then, overwinter. All field studies and material samplings were conducted at the Omoide gawa study site, Taka-cho (35° 06′ N, 134° 55′ E, 190–230 m in altitude) [30] except for field transcriptome data that were previously collected at the Monzen site [33]. The Monzen site (35°05′ N, 134°54′ E, 140–150 m in altitude) is located 3.5 km from the Omoide gawa site [30]. On both sites, *A. halleri* plants grow in open places along small streams running through secondary forests and plantations of *Cryptomeria* and *Chamaecyparis*. The TuMV-infected plants were 57% of examined *A. halleri* plants as sown by a previous plot census [31]. Daily mean temperature records during study periods were obtained from the nearest meteorological station of AMEDAS (Automated Meteorological Data Acquisition System, Japan Meteorological Agency) at Nishiwaki (ID 63331, 34° 59.9′ N, 134° 59.8′ E, 72 m in altitude).

### Detections of TuMV from 3-year time-series samples

The sampling of 3-year time-series observations on TuMV dynamics was performed using leaves obtained from clonal patches in which genetically identical rosettes were aggregated. In September 2012, we identified two TuMV-infected plant patches in the natural habitat of *A. halleri* (designated as plant patches 1 and 2). As plant patch 2 was heavily damaged by deer herbivory in January 2014, we further identified another infected patch (plant patch 3). Samples from plant patch 1 were obtained for the whole observation period (157 weeks from September 2012 to September 2015); however, samples from plant patches 2 and 3 were obtained for shorter periods (68 weeks between September 2012 and January 2014 and 67 weeks from April 2014 to September 2015, respectively). On each sampling day, we collected one fully expanded young leaf (ca. 1 cm in length of leaf blade) from a randomly chosen rosette within the plant patches. Each leaf was sampled by a tweezer (sterilised with 70% ethanol prior to each sampling) and was immediately placed in a 1.5 mL microtube with 1.0 mL RNAlater (Ambion, Life Technologies, Carlsbad, CA, USA) to protect RNA degradation. The samples were kept on ice and were brought back to the laboratory. The samples were immersed in RNAlater at 4 °C for 1 day, and then stored at –20 °C until further analysis.

To estimate TuMV level, we conducted RT-qPCR for the samples from three infected patches every 2 weeks during their observation periods (77, 31, and 38 samples in total for plant patches 1, 2, and 3, respectively, Supplementary Table S1). As a control, we also conducted RT-qPCR on a putative uninfected patch (based on the results from RNA-Seq) that existed for the entire period (38 times in total) and confirmed that no TuMV was detected in the plant patch (data not shown). To estimate the levels of virus replication, strand-specific RT-qPCR were conducted every month (37, 17, and 18 samples in total for plant patches 1, 2, and 3, respectively, Supplementary Table S1). Seasonal changes in the TuMV accumulation were fitted by smooth spline. Value of spar was set at 0.5. The detailed procedure for RNA extraction, RT-qPCR, strand-specific RT-qPCR, and RNA-Seq analyses are provided in the Supplementary methods.

### Consensus sequence and phylogenetic analysis of TuMV

To evaluate the temporal constancy of TuMV genome sequences within single plant patches during the 3-year observation period, consensus TuMV sequences were determined by RNA-Seq for selected samples (Supplementary Table S1, DRA008908). We used the TuMV sequence of NCBI UK1 as the reference for mapping RNA-seq data. Consensus sequences were obtained using samtools and bcftools, with the criteria of mapQ score > 10 and depth ≥ 3. SNP variation within a sample was very low; there was no variation for majority of the SNPs, and if any, the proportion of dominant base was greater than 96.5%. For phylogenetic analyses, samples which showed more than 90% of covered rate of the TuMV reference sequence were selected. Consequently, 17, 14, and 6 TuMV sequences were obtained from plant patches 1, 2, and 3, respectively (Supplementary Table S1). We also included a one-time sample from six infected plants located distantly from the three plant patches and from each other in order to compare TuMV sequences within and between host plant patches. After the incorporation of 14 reported sequences, the sequences were aligned and subjected to a phylogenetic analysis using the Neighbour-Joining method by MEGA 6.06, with the bootstrap of 1,000 [34]. The sequence of TuMV basal-B strain (IRNTRa6; whose original host is *Rapistrum rugosum*) [35, 36] was designated as a outgroup. All positions containing gaps and missing data were eliminated, and the remaining 7,283 bases were used. In addition, *H*, θ, and *π* were calculated using DnaSP [37].

### Seasonal patterns in TuMV distribution within plants

The TuMV distribution in leaves at different positions within plants was examined in the natural population to determine whether winter decrease in virus occurs in the whole plant or only in newly expanded leaves. The TuMV amount was quantified by RT-qPCR. The four sampling times were set at 2-month intervals on 22 December 2015 and 23 February, 19 April, and 21 June 2016. Sampled plants were at the rosette phase in December and February, in the flowering phase in April, and with newly formed rosettes in June. At each sampling time, we sampled four infected plants (Supplementary Table S2). To select these plants, we first collected the oldest leaf from each of 23–40 tagged plants that were separated at least 1 m in distance 1- to 2-week prior to each sampling date and identified TuMV-infected plants by RT-qPCR. On each sampling date, we randomly selected four plants from the infected plants, and harvested whole plants. Sampled plants were kept in plastic bags with ice and brought back to the laboratory. We also collected three uninfected plants on each sampling date as controls.

For all sampled plants, we harvested eight leaves at different positions/plants and preserved them separately in 1.5 mL microtubes with 0.8 mL RNAlater within 48 h after sampling. We numbered positions of leaves acropetally from basal leaves along the main stem as follows: positions 1 and 8 represent the lowest and uppermost leaves (>2.5 mm in length), respectively. The number of leaves per plant ranged from 6 to 17. When the leaf number was 9‒17, every other leaf was sampled at lower positions to make the total number of sampled leaves eight. When the leaf number was less than eight (one plant), lower positions were treated as missing. Leaves in RNAlater were kept at 4 °C for 1 day, and then stored at –20 °C until analysis. The TuMV quantifications were conducted by RT-qPCR. We applied generalised linear model (GLM) with an underlying Gaussian distribution and log link function to evaluate difference in TuMV accumulation across leaf positions. The full model included effects of position, sampling month, and interaction between position and month. The model were compared those with position and month effects and with position effect only. The best model was selected using Akaike’s information criteria. For the month for which the interaction term was significant in the GLM analyses, we further conducted multiple comparisons to TuMV accumulation at different leaf positions for each month using Bonferroni corrected paired *t*-tests (two-sided), to evaluate in which positions the level of virus accumulation differed. All statistical analyses, including those described later, were conducted using R 3.4.1 software [38].

### Temperature-switch experiment

We conducted a 12-week temperature-switch experiment to examine the temperature dependency of TuMV spread into newly formed leaves of infected plants. We collected three infected plants at the Omoide gawa site and used them to multiply infect clonal rosettes. The plants were incubated in a warm growth chamber to enhance bolting (20 °C/15 °C, day/night at 12 h day-length) for 35 days. Then, a 16 h day-length was employed to promote flowering and clonal rosette formation. After the clonal rosettes produced roots, rosettes were transplanted into new pots with soils, a 1:1 mixture of pumice (Kanuma pumice, fine-grained) and culture soil (*N*, *P*, *K* = 320, 210, and 300 mg/L, Takii, Japan). We obtained eight, four, and four clonal rosettes from the original three infected plants, respectively. These infected plants were incubated for ca. two months in a growth chamber (25 °C/15 °C, 12/12 h) until they were used for the experiment. The plants were divided into two groups, with eight plants (four, two, and two from the original three plants, respectively) per group, to apply following two temperature treatments.

We set two temperature treatments in which plants were subjected to either high (25 °C/15 °C) or low (15 °C/5 °C) temperatures for the first 42 days, and then, alternate temperatures for the following 42 days using two growth chambers (NC-220SZ, Nksystem). We named the two treatments as H-L and L-H conditions. Day-length was 12 h for both conditions. Light was supplied by fluorescent lights for plant growth (BIOLUX-A, NEC, Japan). In addition, PAR and R:FR at the pot-surface level for the H-L and L-H growth chambers were averaged to 115.0 ± 9.9 μM/s/m^2^ and 3.20 ± 0.11, and 115.4 ± 9.9 μM/s/m^2^ and 3.18 ± 0.11, respectively. During the experimental period between day 0 (start of the two treatments) and 84 (end of the treatments), we recorded total number of leaves (withered leaves were excluded) once a week for all plants to calculate relative increase rates of leaves. Relative increase rates of leaves were estimated as slopes of regressions of natural logarithm of leaf number on experimental days. These values were obtained for each high- and low-temperature condition for the first 42 days and the following 42 days separately. On day 0 and 84, leaf samples were obtained from all plants. At other sampling times, leaves were sampled from half of the investigated plants (i.e. at day 15, samples were taken from half of the plants, and they were also sampled at days 42 and 70. Leaves were sampled from the remaining half of the plants on days 28 and 56). During each sampling time, we sampled the first and second newer leaves to quantify TuMV level. The leaves were preserved in RNAlater and kept at 4 °C for 1 day. The samples were stored at –20°C until they were analysed. The TuMV quantifications were conducted by RT-qPCR. For each of measurement dates, the difference in TuMV accumulation in the leaves between the treatments was examined by the Mann–Whitney *U* test. The effects of temperature on relative increase rate of leaf were also examined by the Mann–Whitney *U* test.

### Seasonal transcriptome analysis on the effects of TuMV infection

We compared transcriptomes of TuMV-infected and -uninfected plants using data obtained during four different seasons at the Monzen site in our previous study (DRA005873, DRA005874, and DRA005875) [33]. Although the samples were originally collected randomly, they turned out to include 27.4% TuMV-infected plants on average. In 2013, 48 h samplings (starting from 16:00 on the first day) were performed at the spring equinox (19–21 March), the summer solstice (26–28 June), the autumn equinox (24–26 September), and the winter solstice (12–14 December). For each season, we used transcriptome data from 48 plants from different plant patches. Young expanded leaves were sampled from these plants during the sampling period (two plants/24 timings at 2-h interval). We excluded two and four samples for SS and AE, respectively, which had less than 10^5^ total reads. We mapped the RNA-seq reads on TuMV and distinguished infected and uninfected plants. The number of infected/uninfected plants were 12/36, 13/33, 13/31, and 13/35, for SE, SS, AE, and WS, respectively. There was no apparent bias in the sampling time between the infected and uninfected plants. Chloroplast-coded genes, genes with averaged mapped reads < 10, and genes with unmapped samples > 10 were excluded from the analysis of each season. Our previous study reported occurrence of other viruses, i.e., *Cucumber mosaic virus* (CMV), *Brassica yellow virus* (BrYV), and *Arabidopsis halleri partitivirus 1* (AhPV1), in the study area [31]; however, the first two viruses cannot be detected by the polyA-targeted procedure used for the RNA-seq in this study. Because the sampling was conducted under natural conditions, we grouped TuMV-infected and -uninfected samples solely by the presence/absence of TuMV without controlling other pathogens. The differentially expressed genes (DEGs) were identified between infected and uninfected plants from the remaining 15,714, 15,597, 16,026, and 10,273 genes using EdgeR [39] for SE, SS, AE, and WS, respectively. Multiple testing corrections were performed by setting the FDR (false discovery rate) [40]. Gene ontology (GO) analyses were applied for these DEGs using the GO.db package from the Bioconductor project for the R 3.1.1 software following the method described in our previous study [33], and we used an updated GO list for the *A. halleri* transcriptome by applying GO terms for *A. thaliana* (TAIR10, http://www.arabidopsis.org/) to sequence homologues in *A. halleri*.

## Results

### Long-term dynamics of within-host TuMV accumulation

**Fig. 1:**
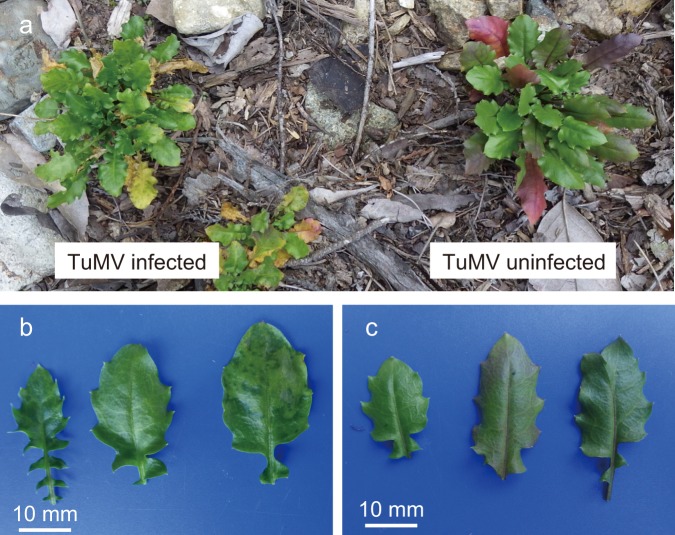
TuMV-infected and uninfected plants in a natural population of *Arabidopsis halleri*. **a** An example of infected and uninfected plants in which symptomatic differences are clear. Leaves of the infected plants with mosaic symptoms (**b**) and those of uninfected plants (**c**). The images were captured in early November

**Fig. 2:**
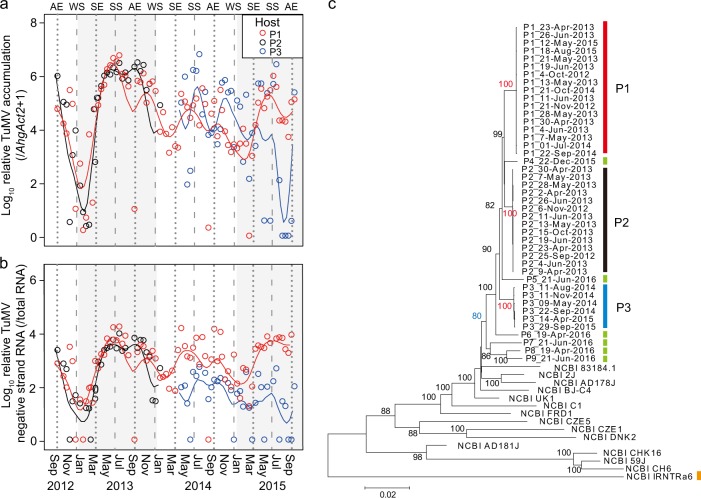
Three-year observation on within-host accumulation and phylogenetic relationship of TuMV in three infected *Arabidopsis halleri* plant patches. Temporal changes of accumulation (**a**) and negative-strand RNA (**b**) of TuMV in upper leaves of plant patches 1, 2, and 3 (P1, P2, and P3, respectively). Solid lines indicate smooth-spline fittings. Negative-strand RNA was detected by strand-specific RT-qPCR. Broken lines and dashed lines indicate spring/autumn equinoxes (SE/AE) and summer/winter solstices (SS/WS), respectively. The year periods (i.e., 2012, 2013, 2014, and 2015) were shown by white and grey shadings. **c** Phylogenetic relationships between TuMV strains detected from the three patches (red, black, and blue bars represent P1, P2, and P3, respectively) during the 3-year observation. One-time sample from additional six plants were included (P4‒P9; indicated by green coloured bars). Sample IDs were coded by plant patches and sampling dates. Previously reported five, four, and four strains selected from Japan, continental Asia, and Europe, respectively, were included, and the sequence of TuMV basal-B strain (IRNTRa6; whose original host is *Rapistrum rugosum*) was used as an outgroup (indicated by an orange coloured bar). The bootstrap values (% of 1000 replicates) are listed next to the branches. The bootstrap values in red supported the monophyly of virus sequences within plant patches 1, 2, and 3, and the value in blue supported the monophyly of virus sequences from the studied population

To examine whether persistent viral infections exist in natural plant populations, we performed 3-year biweekly monitoring of TuMV for three infected plant patches. The TuMV-infected plants showed normal growth and mosaic symptoms were minor in most plants (Supplementary Fig. S1). Some infected plants occasionally developed mosaic symptoms, especially during autumn (Fig. 1). During the 2012‒2013 winter, TuMV accumulation in plant patches 1 and 2 decreased remarkably, and then, increased during spring by ~10^4^‒10^5^ fold (Fig. 2a). Winter decrease in TuMV level was also observed during 2013‒2014 and 2014‒2015 winters for plant patch 1; however, the patterns were less conspicuous (Fig. 2a). We detected significant positive correlations between TuMV accumulation and daily mean temperature of the sampling date for plant patches 1 and 2 (Supplementary Fig. S2). The amount of negative-strand RNA, an indicator of virus replication activity [41], showed temporal patterns similar to those of TuMV accumulation (Fig. 2b). The similarity was supported by significant correlations between TuMV accumulation and its negative-strand RNA across the observation period for all infected patches (correlation coefficients ≥ 0.80, *P* < 0.001; Supplementary Fig. S3).

### Temporal constancy of TuMV sequences within hosts

To evaluate temporal constancy of TuMV genome sequences within individual plant patches during the 3-year observation period, consensus TuMV sequences for selected samples were compared. In the 9,495 bp of the total TuMV genome, 6,919 bp were possible to compare across all 37 samples obtained from the three plant patches in this study, and the total number of mutation sites were 142 (2.07%) in the region. Among the 142 SNP sites, 116 differed between patches but were fixed within patches. Virus sequences obtained from each of the three plant patches formed a cluster for each host genotype (Fig. 2c). We observed very low levels of TuMV SNP variation within plant patches (nucleotide polymorphism *θ* = 2.6 × 10^−4^–5.8 × 10^−4^, nucleotide diversity *π* = 1.2 × 10^−4^–5.3 × 10^−4^) relative to those among different plant patches (*θ* = 2.9 × 10^−2^, *π* = 2.4 × 10^−2^; Supplementary Table S3). These results indicated that a specific strain of TuMV was dominant in each infected plant patch throughout the observation period.

### Seasonal patterns of TuMV distribution within plants

**Fig. 3:**
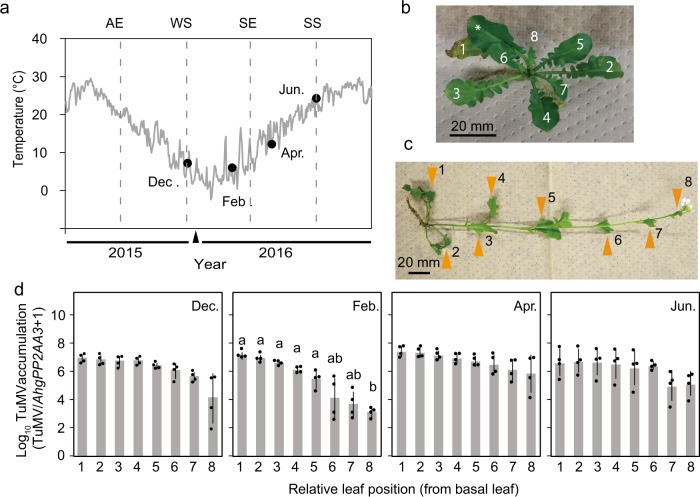
Suppression of TuMV accumulation in upper leaves of *A. halleri* during winter. **a** Annual temperature regimes of the study site and temperatures on the sampling dates. The grey line represents daily mean temperatures obtained from the nearest meteorological station (see “Materials and methods” section). Closed circles represent four sampling dates set at 2-month intervals, i.e. 22 December 2015, and 23 February, 19 April, and 21 June 2016. Dashed lines indicate spring/autumn equinoxes (SE/AE) and summer/winter solstices (SS/WS). A triangle on the *x*-axis represents 1 January 2016. **b** Example of numbering leaf positions in an infected rosette. Positions 1 and 8 correspond to the lowest and uppermost leaves (>2.5 mm in length), respectively. When the leaf number was more than eight, every other leaf was sampled at lower positions to make the total number of sampled leaves eight (an asterisk indicates a skipped leaf). **c** An example of leaf positions in infected flowering plants in April. Although the stem was elongated, rosette and cauline leaves along the main stem (indicated by orange triangles) were harvested. Positions 1 and 8 correspond to the lowest and uppermost leaves, respectively. **d** The TuMV level in leaves at different positions within plants examined at the four sampling dates in the natural population. Rosettes (December and February), flowering plants (April), and newly formed aerial rosettes (June) were examined. Averages and standard deviations are shown. Different letters above bars indicate significant differences in pairwise comparisons using paired *t*-tests (two-sided, *P* < 0.05, diagram-wise significance levels were adjusted using Bonferroni correction), while *NS* represents no significance for all combinations

The TuMV distribution was examined four times in two month intervals between WS and SS with different temperature regimes (Fig. 3a). Plants were in the rosette stage in December and February (Fig. 3b). They were in the reproductive stage in April, and leaves were collected along the elongated flowering stems (Fig. 3c). By June, most of the leaves along with the stem had withered and new clonal rosettes were formed from lateral meristems; we sampled leaves from these new rosettes. The accumulation of TuMV in the upper leaves was distinctively lower than that in the basal leaves in February (Fig. 3d). As a result of GLM, the model including leaf position, month and their interaction terms were selected, and the effect of leaf position was highly significant in February (Supplementary Fig. S4). The virus level was 1/11,400 in the top most upper leaves (position 8) relative to the basal leaves (position 1) in February and the difference was statistically significant (Fig. 3d). The virus was abundant across all leaf positions along flowering stems in April, and in all leaves of new clonal rosettes in June (Fig. 3d). In June, the clonal rosettes were in the stage of aerial rosettes, and thus, clonal transmission was likely to occur through stems of mother plants. The results indicated that the decrease in TuMV level occurs in the newly formed leaves during winter; however, virus preserved in the lower leaves spread into the upper leaves in spring and subsequently into new clonal rosettes.

### Temperature dependency of TuMV spread within plants

**Fig. 4:**
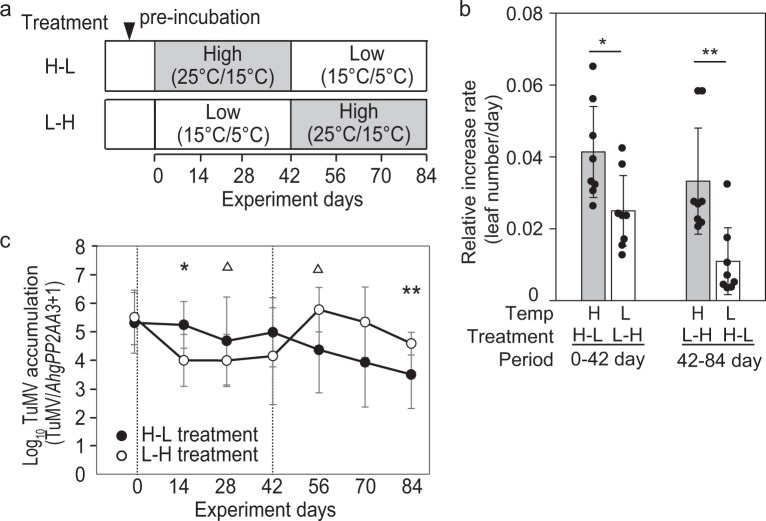
Suppression of TuMV accumulation in upper leaves by low temperature in the temperature-switch experiment. **a** Experimental design showing temperature conditions for H-L and L-H treatments. Plants were exposed to either high (25 °C/15 °C) or low (15 °C/5 °C) temperatures for the first 42 days and then alternate temperatures for the following 42 days. **b** Relative increase rate of leaf number under low- and high-temperature conditions during 0‒42 and 42‒48 days (*N* = 8). Averages and standard deviations are shown. Asterisk and double asterisk represent significant differences at *P* < 0.05 and *P* < 0.01, respectively, in the Mann–Whitney *U* tests. **c** Temporal changes in TuMV accumulation for the H-L and L-H treatments during the experiment. Averages and standard deviations of TuMV accumulations at the third newer leaves were shown. Triangle, asterisk, and double asterisk represent significant difference between treatments at *P* < 0.07 (marginal significance), *P* < 0.05, and *P* *<* 0.01, respectively, in the Mann–Whitney *U* tests

In the temperature-switch experiment using infected plants (Fig. 4a), new leaves were formed under both high and low temperatures, and the rate of leaf production was temperature dependent (Fig. 4b). Significantly higher rates of leaf increase were detected under the high-temperature regime during the first period (0‒42 days, Fig. 4b). After temperature conditions were switched (42‒84 days), leaf increase rate was reversed, and the difference was statistically significant. Virus accumulation in the newly formed leaves was temperature dependent (Fig. 4c). In the H-L treatment, the virus accumulation was kept high until day 42 but was decreased in the successive low-temperature conditions (Fig. 4c). In the L-H treatment, the virus amount decreased during the low-temperature conditions but increased in the successive high-temperature conditions (Fig. 4c). The differences in virus accumulation were statistically significant (*P* < 0.05) on days 14 and 84 and were marginally significant (*P* < 0.07) on days 28 and 56. The production rate of leaves was low, and newly formed leaves showed less accumulation of TuMV under low-temperature conditions, which corresponded with winter decrease of virus accumulation in the natural population.

### Seasonality in transcriptomic difference between TuMV-infected and -uninfected plants

**Fig. 5:**
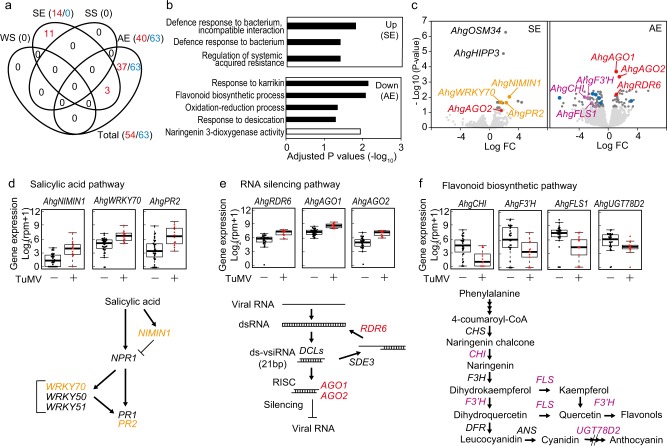
Season dependent differentially expressed genes in the transcriptome analyses of differences between TuMV-infected and -uninfected plants on spring/autumn equinoxes (SE/AE) and summer/winter solstices (SS/WS) in the natural population of *A. halleri*. **a** Venn diagrams illustrating the overlap in differentially expressed genes between infected and uninfected plants (DEGs, adjusted *P* < 0.05) in the four seasons. Red and blue numbers represent up- and down-regulation in infected plants. **b** Adjusted *P* values for enriched GO terms (*P* < 0.05) in DEGs upregulated in infected plants in SE and those downregulated in infected plants in AE. Black and white bars represent GO terms in biological process and molecular functions, respectively. All of the DEGs were upregulated in SE, and there was no enriched GO in upregulated DEGs in AE. **c** Volcano plots between infected and uninfected plants on SE and AE. Up- and down-regulated genes in infected leaves show positive and negative log_2_ fold change, respectively. The DEGs related to salicylic acid-dependent defence, RNA silencing, flavonoid biosynthetic process, and oxidation-reduction process are shown by orange, red, purple, and blue dots, respectively. Representative genes are also listed. The dark grey dots represent other DEGs. **d** Examples of gene expression for defence-related SE DEGs. Homologues of DEGs in the salicylic acid-dependent defence pathway are shown by orange letters. **e** Examples of gene expression for defence-related AE DEGs. Homologues of DEGs in the RNA silencing pathway are shown by red letters. ds-vsiRNA, double stranded virus-derived small interfering RNA; RISC, RNA-induced silencing complex. **f** Examples of AE DEGs related to flavonoid biosynthetic process. Homologues of DEGs related to the flavonoid biosynthetic pathway are shown by purple letters. CHS chalcone synthase, CHI chalcone isomerase, F3H flavanone 3-hydroxylase, F3′H flavonoid 3′-hydroxylase, FLS flavonol synthase, DFR dihydroflavonol 4-reductase, ANS anthocyanidin synthase, UGT78D2 anthocyanidin 3-O-glucosyltransferase. In **d**–**f**, gene expressions of uninfected (TuMV−) and infected (TuMV+) plants were shown by black and red circles, respectively. Mean, one and three quartiles, and 95% confidence limits are shown by box plots

We compared gene expression between infected and uninfected plants, and identified DEGs in each season (referred to as SE DEGs, SS DEGs, AE DEGs, and WS DEGs; Supplementary Table S4). For all seasons, samples were collected at 2-h intervals for 48 h and there was no apparent bias in the sampling time between the infected and uninfected plants (Supplementary Fig. S5). The transcriptomic differences were most prominent in AE DEGs (103 genes, Fig. 5a). We found 14 SE DEGs and no DEGs for SS and WS (Fig. 5a). Furthermore, SE and AE DEGs were mostly specific to each season, and three DEGs, homologues of *AGO2*, *PHLOEM PROTEIN 2-B1* (*PP2-B1*), and *IST1-LIKE 6* (*ISTL6*), were common between SE and AE (Fig. 5a). In *A. thaliana*, AGO2 is known to be the most important argonaute protein for antiviral RNA silencing in leaves against TuMV infection [42], and PP2-B1 is a phloem protein 2 [43] that plays a role in the establishment of phloem-based defence mechanisms against aphid infestation [44, 45]. Moreover, SE DEGs were characterised by upregulation in infected plants for all 14 genes, while AE DEGs showed both upregulation and downregulation (40 and 63 genes, respectively) in infected plants (Fig. 5a). Three significantly enriched GOs for SE DEGs were all defence-related ones, and these GOs included genes that also served as an anti-virus defence [46, 47] (Fig. 5b; Supplementary Table S5). The significantly enriched GOs were detected only for downregulated DEGs in AE, and they included responses to karrikin, flavonoid biosynthetic process, oxidation-reduction process, response to desiccation, and naringenin 3-dioxygenase activity (Fig. 5b; Supplementary Table S5). Upregulation of defence-related genes in infected plants was detected both on SE and AE, but a different set of genes in distinct defence mechanisms were identified (Fig. 5c). In SE, homologous genes of the salicylic acid (SA) dependent defence, which is associated with local and systemic acquired resistance [46–50], were upregulated, including homologues of *NIM1-INTERACTING 1* (*NIMIN1*), *WRKY 70* (*WRKY70*), and *PATHOGENE-RELATED PROTEIN 2* (*PR2*, Fig. 5d). While in AE, genes related to RNA silencing [51, 52], such as homologues of *RNA-DEPENDENT RNA POLYMERASE 6* (*RDR6*), *AGO1*, and *AGO2*, were upregulated (Fig. 5e). Some of these defence-related DEGs oscillated diurnally (e.g. *AhgRDR6*, Supplementary Fig. S6), but such diurnal oscillation pattern did not affect detection of season specific DEGs (the diurnal patterns were shown for homologues of *NIMIN1*, *WRKY70*, *PR2*, *RDR6*, *AGO1*, and *AGO2* in Supplementary Fig. S6). Genes that downregulated in infected plants on AE included those related to flavonoid biosynthesis (Fig. 5f). Some flavonoids in the pathway are phytoalexins that exhibit anti-fungal and anti-virus activities, and the downregulation of flavonoid biosynthesis genes has been considered as a counter-defence by pathogens [53–55]. The downregulation of these genes in AE coincided with the observation that TuMV-infected plants lacked the accumulation of anthocyanin on leaves, which were commonly observed in uninfected plants in autumn (Fig. 1a). Overall, we detected activation of distinct defence responses in host plants in spring and autumn, while transcriptomic difference, other than defence genes, appeared transiently during autumn.

## Discussion

The seasonal schedule of TuMV replication and seasonal growth cycle of *A. halleri* allows both successful clonal propagation of the host and stable viral transmission to host clonal offspring. In the TuMV–*A. halleri* system, transcriptomic difference other than defence-related genes appeared only in autumn, being represented by the largest number of DEGs across seasons. Autumn symptoms are expected to be transient until temperature decreases. Low temperature suppressed the speed of virus spread/replication, and consequently, kept virus accumulation low in newly formed leaves during winter. It is likely that transient symptoms and reduced within-host virus accumulation provided by winter temperatures allowed the persistent host growth. The temperature dependence of TuMV spread within plants observed here was consistent with that in previous experimental studies [19]. On the other hand, lower leaves of winter rosettes functioned as a reservoir for the virus that subsequently transmitted to the clonal offspring of the host in spring, which resulted in high transmission rate (92%) as we reported previously [32]. Our study is the first to report seasonality in viral dynamics and virus–host interactions in naturally occurring infections of plant viruses. Although the importance of seasonality in determining virus–host interaction has been recognised widely [56, 57], it has been primarily studied in terms of population-level infection rate and vector activity [12, 13, 57]. For example, population-level TuMV infection rates have been reported to peak during early growth stages in March in natural *A. thaliana* populations in central Spain [4].

Interestingly, distinct defence mechanisms have been suggested to function in spring and autumn. In March, we observed upregulation of homologous genes related to the SA dependent defence in TuMV-infected plants, and SA has been considered to play a critical role in plant innate immunity [58]. In spring, symptom at the level of transcriptomes was weak; limited to 14 defence-related genes. This is probably because the virus in the young leaves is in its initial phase of increment in response to spring warm temperature (Figs. 2 and 3), and because these leaves are defended by local SA dependent defence that may be triggered systemically by SA signals from older leaves with high virus accumulations. SA dependent systemic acquired resistance has been reported to be the first defence mechanism activated in response to plant virus infection [59]. As the activation of SA dependent defence can antagonise jasmonic acid (JA) dependent defence [60, 61], which is known to be a major defence mechanism against insect vectors [62], it is interesting to see whether SA-JA relationship is conserved in *A. halleri* and whether the activation of SA dependent defence in spring consequently enhance horizontal transmission via aphids through the JA suppression. Increased vector attraction by virus infection through the SA-JA antagonistic relationship has been reported for Tospovirus-infected *A. thaliana* [63]^.^ In autumn, when within-host virus accumulation was high, we detected an upregulation of homologous genes involved in RNA silencing, which is a defence mechanism against viruses [26, 28] and other infectious organisms [64]. This finding suggests that direct defence against accumulated virus became more important in autumn. Our results raised a question how host plants shift their defence strategies seasonally in response to natural dynamics of viruses and other factors. Another autumn specific response in defence-related pathways was the downregulation of some homologues in the flavonoid biosynthetic pathway, which may represent a counter-defence by the accumulated virus. Flavonoids produced by the pathway, such as naringenin, kaempferol, and quercetin, have been reported to exhibit antimicrobial activity [65–68]. The concentration of kaempferol was reportedly lowered in the upper leaves of *A. thaliana* by the experimental infection of *Cucumber mosaic virus* with satellite RNA [55], and this corresponds with the lower expression of *AhgFLS* in TuMV-infected plants in our observation in autumn. The downregulation of these genes in AE coincided with the observation that TuMV-infected plants lacked the accumulation of anthocyanin on leaves which were commonly observed in uninfected plants in autumn (Fig. 1a). Because anthocyanin is considered to have anti-oxidative activity and UV-B protective function [69], the reduced flavonoid accumulation may influence tolerance of TuMV-infected plants against these abiotic stresses. We detected no DEGs in SS despite of high TuMV accumulation. In our previous study in which transcriptomes of infected and uninfected plants were compared once in equivalent season, we also detected small number of DEGs [31]. One explanation is existence of abiotic/biotic determinants of gene expressions other than TuMV. Another explanation is that either host response or virus virulence became weak in the period. Further experimental studies are required to identify the causal factors for no DEGs in SS.

It has been speculated that long-term pathogen–host interactions via vertical transmission favours low viral virulence and high host resistance [70–73]. However, TuMV is transmitted horizontally via aphids [74], and vertical transmission through seeds is rare [75, 76]. In the TuMV  – *A. halleri* system, the effective transmission of virus through clonal propagation [32] is likely to provide a similar situation that favours low viral load to host plants as long as a single virus lineage utilises its own host for longer periods. Actually, it turned out that a single unique lineage of TuMV was able to persist for over a year in a single clonal lineage of host plants, being transmitted through clonal propagation of *A. halleri*. In one case, we confirmed that a single lineage of TuMV remained within a clonal plant patch for 3 years and probably longer. Low sequence variation of TuMV genome within clonal patches of *A. halleri* relative to that between patches suggested that the horizontal transmission rate was extremely low making subsequent infection of TuMV rare. Otherwise, the mechanism for cross protection, the ability of an established virus to interfere with second infection by other strains of the same virus [77, 78], might be present. A specific amino acid substitution in helper component-proteinase (HC-Pro) in a TuMV strain has been reported to confer cross protection against another strain [79]; however, the TuMV strains in our study site do not possess this particular amino acid substitution. It is expected that cross protection by mild strains that have less effect on host fitness will enhance the persistent infection via exclusion of severe strains. Whether co-evolution between plant virus strains and host genotypes occurs in linage-to-linage persistent infection is an open question to be asked in future studies.

As our results suggested, the processes that keep viral load low were highly season dependent in a natural environment. We predicted that changes in the temperature regime might lead to imbalances in the virus–host interactions identified here. Further long-term field and experimental studies are required to evaluate how the seasonal processes are important in maintaining persistent relationships between virus and host individuals. Our study demonstrated that the combined approach to evaluate field situations and to reproduce the expected effects in the laboratory provides a robust strategy to study host–virus interactions as a part of ecosystem. Remaining questions to be challenged in future studies include effects of endogenous environments, such as crosstalk with clock genes [80] and interaction with other infectious organisms coexisting with TuMV [30]. As seasonality in virus–host interactions partly depends on temperature, future climate change, such as global warming, is likely to alter distribution of native viral infection in natural plant communities.

## Supplementary information

Supplementary Materials and Methods

Supplementary Figures

Supplementary Tables
