# Supplementary material for: Seasonality of interactions between a plant virus and its host during persistent infection in a natural environment
Source: ISME J. 2019 Oct 30;14(2):506–18. doi: 10.1038/s41396-019-0519-4 (PMC6976672; doi:10.1038/s41396-019-0519-4)
Supplement: Supplementary file 1 — Supplementary Materials and Methods [file 41396_2019_519_MOESM1_ESM.pdf]

Supplementary Materials and Methods

Seasonality of interactions between a plant virus and its host during persistent infection in a natural environment

Mie N. Honjo, Naoko Emura, Tetsuhiro Kawagoe, Jiro Sugisaka, Mari Kamitani, Atsushi J. Nagano, and Hiroshi Kudoh

RNA-extraction.

In the leaf samples from the three-year time-series observation, total RNA was extracted using an RNeasy Plant Mini Kit (Qiagen, Hilden, Germany) without DNase treatment, according to the manufacturer’s instruction. For RT-qPCR, the extracted RNA samples were treated with DNase I (Takara, Tokyo, Japan) and then purified using AMpure XP beads (Beckman, Brea, CA, USA). For RNA-Seq, the extracted RNA samples were used directly for library preparations. In other samples, total RNA was extracted with a Maxwell® 16 instrument using Maxwell® 16 LEV Plant RNA kit according to the manufacturer’s instructions (Promega, Madison, WI, USA). The leaves were completely crushed with cylinder-shaped metal beads using Multi beads shocker (Yasui Kikai, Osaka, Japan). The amount of RNA was measured by Qubit Fluorometer using Quant-iT™ RNA Assay Kit (Life Technologies) or by Quantus Fluorometer using QuantiFluor® RNA System (Promega), and the quality was assessed using a Bioanalyzer (Agilent Technologies, Santa Clara, CA, USA).

RT-qPCR.

To quantify TuMV amount in leaves, RT-qPCR was performed by the following procedure. Total RNA (200 ng) was reverse transcribed in a 20 μL reaction (High-

Capacity cDNA Reverse Transcription Kit, Life Technologies) with oligo dT primer according to the manufacturer's instructions. The qPCR was performed with 1μL of the cDNA using the QuantStudio 7 (Applied Biosystems, Foster City, CA, USA). The amount of TuMV RNA was calculated relative to a pre-prepared standard cDNA sample which we included in all qPCR trials. Primers TuMVCP-F (5′-TGGCTGATTACGAACTGACG-3′, designed here) and CP-R (5′-CTGCCTAAATGTGGGTTTGG-3′)[1] were used for TuMV detection. We used *AhgActin2* and *AhgPP2AA3* as reference genes for normalization of the three-year time-series samples and other samples, respectively. Primers of these genes were AhgACT2F: 5′-TCCCTCAGCACATTCCAGCAGAT-3′ and AhgACT2R: 5′-AACGATTCCTGGACCTGCCTCATC-3′[2] and AhgPP2AA3-F: 5′-GTATGCACATGTTTTGCTTCCAC-3′ and AhgPP2AA3-R: 5′-CAACCAAGTCATTCTCCCTCATC-3′[3], respectively. The standard cDNA of TuMV was prepared from total RNA, which was extracted from pooled infected leaves (ca. 10 leaves) of *A. halleri*. The standard cDNA of *AhgActin2* and *AhgPP2AA3* was prepared from pooled un-infected leaves (ca. 10 leaves). Dilution series of the standard cDNA were amplified in duplicate with samples for all qPCR, and we used nine and six dilution levels, i.e., 10^0–10^8 and 5^0–5^5, for TuMV and the reference genes, respectively. Each PCR

reaction contained 1 μL of cDNA solution, 200 nM primers, and Fast SYBR Green Master Mix (Applied Biosystems) in a final volume of 10 μL. The PCR conditions were as follows: 20 s at 95 °C and 40 cycles of 1 s at 95 °C and 20 s at 60 °C. Two technical replicates were prepared for each sample. To evaluate whether PCR products were single or not, a melt-curve was obtained for each sample by a gradual increase of temperature of 0.05 °C/s from 60 °C to 95 °C.

To confirm the replication activity of TuMV, negative strand RNA of the virus was quantified by strand-specific RT-qPCR[4]. We used primers that were designed based on the TuMV sequences at the study site using the Primer Express program (Applied Biosystems). Briefly, 100 ng of total RNA were reverse transcribed in the presence of 250 nM RT primer which specifically anneal to the negative (-) strand RNA; TuMV-I [5′-GGCCGTCATGGTGGCGAATAATACGTGCGAGAGAAGCACACA-3′. Underlined and non-underlined sequences represent nonviral 5′ tag sequences and those homologous to TuMV, respectively] with Superscript III reverse transcriptase (Invitrogen) in 10 μL reactions for 30 min at 55 ˚C. Previous to the reaction, primers were allowed to anneal by incubating 5 min at 70 ˚C and snap cooling on ice. The reverse transcription reaction was stopped by heating at 85 ˚C for 10 min. Specific qPCR was performed in a 10 μL final volume using the Fast SYBR Green Master Mix reagent (Applied Biosystems) with the

same conditions described above. In this specific qPCR, TuMV (-) RNA was quantified from 1 μL cDNA using a set of primers (500 nM for each); i.e., TuMV-II (5′-*AATAAATCATAA*GGCCGTCATGGTGGCGAATAA-3′, underlined sequences are identical with 5′ tag of PII)[4] and TuMV-III (5′-*AATAAATCATAA*ATTTGTTCGGCTTGGATGGA-3′, sequence complementary to TuMV). Primers TuMV-II and TuMV-III contained 5′ flaps (italicized sequence) to improve qPCR[5]. The series of standard cDNA at 1, 1/2, 1/4, 1/8, and 1/16 dilutions were included in all analyses. Two replicates were performed for each sample and standard.

RNA-Seq analysis.

We used previously published RNA-Seq data (accession number; DRA005871, DRA005872, DRA005873) [6], and newly obtained data (accession number; DRA008908) for TuMV detection from the three-year time-series observations (described in detail in main text). For RNA-Seq library preparation of samples from the three-year time-series observations, the modified high-throughput method was used [7]. Quantification of TuMV amount expression from by the RNA-Seq data was conducted as follows[8]. Pre-processing and quality filtering were performed by trimmomatic-v0.32[9]. Virus genome sequences, including TuMV (Complete genome sequences of 3,981 viruses, obtained

from the NCBI GenBank were used as the virus reference sequences.); *A. halleri* transcript sequences (32,648 genes, Dryad Digital Repository, doi: 10.5061/dryad.4pf96)[10]; and ERCC spike in control (Life Technologies) were used as reference sequences. Details of the preparation strategy of the references were described in our previous study[8]. The pre-processed sequences were mapped on the reference and quantified using RSEM-1.2.11[11]. The output of RSEM was analysed using R 3.1.1 software[12]. The TuMV amount was calculated as RPM Calculation of RPM (read per million) values based on the expected count values of RSEM. The number of virus reads was so large that the total read number was very different between infected and non-infected plants. Therefore, we used the total reads derived from host genes, except for rRNA, as a denominator, instead of the total reads including virus reads[8]. The removal of the effects of missorted reads in quantification was performed as described in our previous study[8].

References

1. Wei T, Zhang C, Hou X, Sanfaçon H, Wang A. The SNARE protein Syp71 is essential for *Turnip mosaic virus* infection by mediating fusion of virus-induced vesicles with chloroplasts. *PLoS Pathog* 2013; **9:** e1003378.
2. Aikawa S, Kobayashi MJ, Satake A, Shimizu KK, Kudoh H. Robust control of the

seasonal expression of the *Arabidopsis FLC* gene in a fluctuating environment. *Proc Natl Acad Sci U S A*. 2010; **107:** 11632-11637.

3. Nishio H, Buzas DM, Nagano AJ, Suzuki Y, Sugano S, Ito M *et al.* From the laboratory to the field: assaying histone methylation at *FLOWERING LOCUS C* in naturally growing *Arabidopsis halleri*. *Genes Genet Syst* 2016; **91:** 15-26.
4. Martinez F, Sardanyés J, Elena SF, Daròs J-A. Dynamics of a plant RNA virus intracellular accumulation: stamping machine vs. geometric replication. *Genetics* 2011; **188:** 637-646.
5. Afonina I, Ankoudinova I, Mills A, Lokhov S, Huynh P, Mahoney W. Primers with 5' flaps improve real-time PCR. *Biotechniques* 2007; **43:** 770-774.
6. Nagano AJ, Kawagoe T, Sugisaka J, Honjo MN, Iwayama K, Kudoh H. Annual transcriptome dynamics in natural environments reveals plant seasonal adaptation. *Nat Plants* 2019; **5:** 74-83.
7. Wang L, Si Y, Dedow LK, Shao Y, Liu P, Brutnell TP. A low-cost library construction protocol and data analysis pipeline for Illumina-based strand-specific multiplex RNA-seq. *Plos One* 2011; **6:** e26426.
8. Kamitani M, Nagano AJ, Honjo MN, Kudoh H. RNA-Seq reveals virus-virus and virus-plant interactions in nature. *FEMS Microbiol Ecol* 2016; **92:** fiw176.
9. Bolger AM, Lohse M, Usadel B. Trimmomatic: a flexible trimmer for Illumina sequence data. *Bioinformatics* 2014; **30:** 2114-2120.

10. Kamitani M, Nagano AJ, Honjo MN, Kudoh H. Data from: RNA-Seq reveals virus–virus and virus–plant interactions in nature. *Dryad Digital Repository* 2016; doi: 10.5061/dryad.4pf96
11. Li B, Dewey CN. RSEM: accurate transcript quantification from RNA-Seq data with or without a reference genome. *BMC Bioinformatics* 2011; **12:** 323.
12. R Development Core Team. *R: A Language and Environment for Statistical Computing.* http://www.R-project.org (R Foundation for Statistical Computing, Vienna, 2011).
