# Supplementary material for: Seasonality of interactions between a plant virus and its host during persistent infection in a natural environment
Source: ISME J. 2019 Oct 30;14(2):506–18. doi: 10.1038/s41396-019-0519-4 (PMC6976672; doi:10.1038/s41396-019-0519-4)
Supplement: Supplementary file 2 — Supplementary Figures [file 41396_2019_519_MOESM2_ESM.pdf]

Supplementary figures

Seasonality of interactions between a plant virus and its host during persistent infection in a natural environment

Mie N. Honjo, Naoko Emura, Tetsuhiro Kawagoe, Jiro Sugisaka, Mari Kamitani, Atsushi J. Nagano, and Hiroshi Kudoh

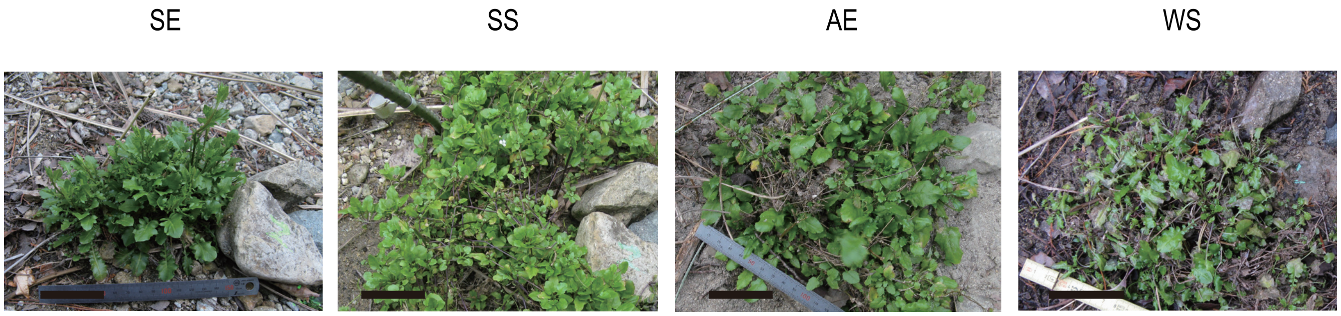

Supplementary Figure S1

Photographs of an infected plant patch of *Arabidopsis halleri* showing typical phenotypes at the spring equinox (SE), summer solstice (SS), autumn equinox (AE), and winter solstice (WS). Scale bars = 5cm.

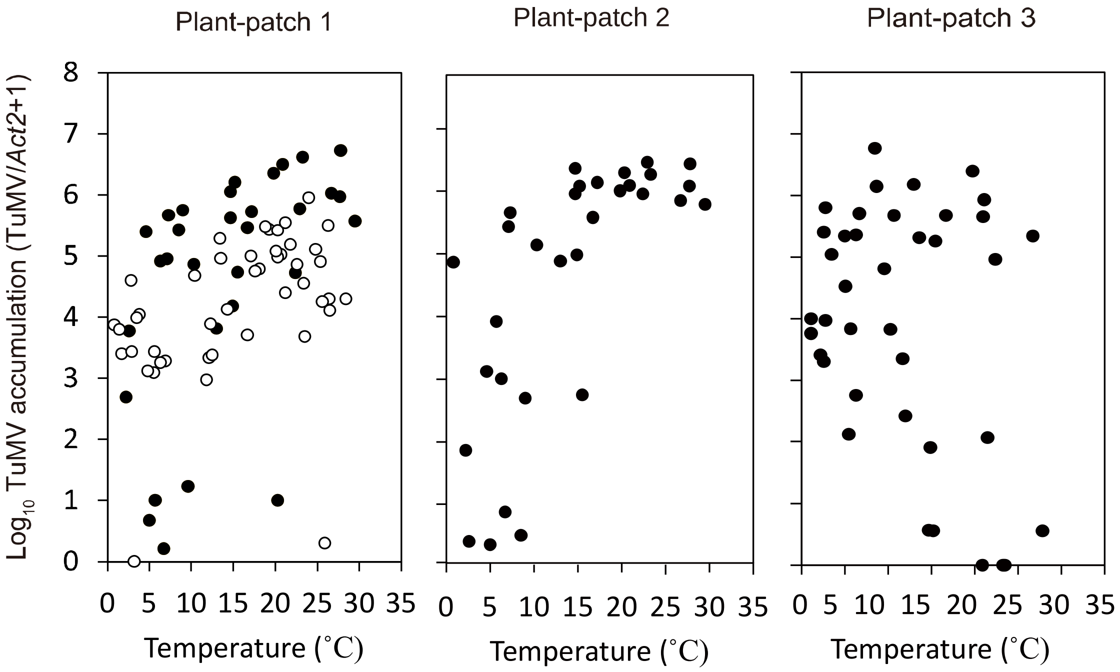

Supplementary Figure S2

The scatter plots between TuMV accumulation in upper leaves and average ambient temperature for three naturally infected patches (plant-patches 1, 2, and 3). TuMV data corresponds to those in Figure 2a. In a panel of plant-patch 1, closed circles and open circles were indicated samples taken in 2012-2013 and 2014-2015, respectively. Spearman's rank correlation coefficient were 0.61, 0.44, and 0.77 for plant-patch 1 in 2012-2013 and 2014-2015, and plant-patch 2 (2012-2013), respectively, and they were significantly different from zero at $P < 0.001$, but no significant correlation ($r = -0.06$, $P > 0.05$) was detected for plant-patch 3 (2013-2014).

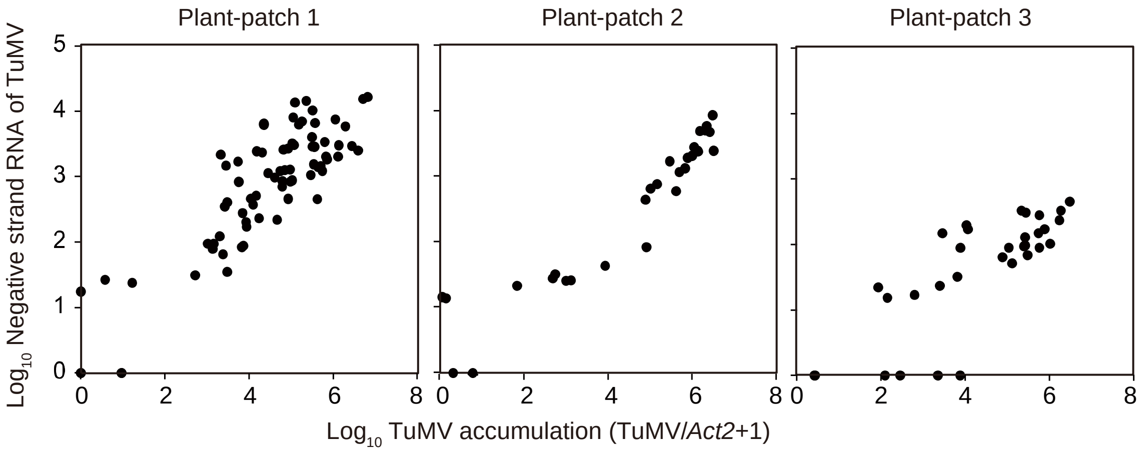

Supplementary Figure S3

The relationship between TuMV accumulation (X axes) and negative strand RNA (Y axes) in upper leaves of three naturally infected patches (plant-patches 1, 2, and 3). Data corresponds to Figure 2a and 2b. Pearson's correlation coefficients were 0.87, 0.95, and 0.80, respectively, and all were significantly different from zero at $P < 0.001$.

(a)

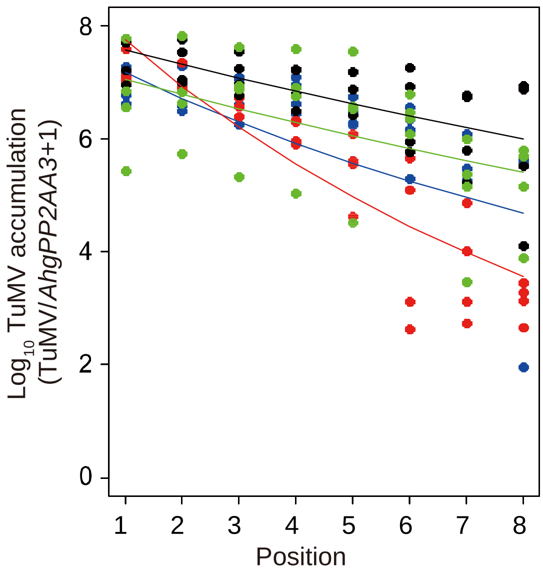

(b)

Coefficients	Estimate	SE	*t*-value	Pr (>|t|)
(A)*glm(log_TuMV ~ Position + Month+ Position:Month family=gaussian(link="log"), data=data_mix)* **AIC: 312.26**				
(Intercept)	2.056	0.043	47.967	<0.001 ***
Position	-0.033	0.009	-3.670	<0.001 ***
Dec	-0.009	0.062	-0.145	0.885
Feb	0.101	0.064	1.591	0.114
Jun	-0.066	0.063	-1.047	0.297
Position:Dec	-0.017	0.013	-1.299	0.196
Position:Feb	-0.078	0.015	-5.239	<0.001 ***
Position:Jun	-0.005	0.013	-0.357	0.722
Null deviance: 203.055 on 127 df, Residual deviance: 74.666 on 120 df				
(B)*glm(formula = log_TuMV ~ Position + Month, family = gaussian(link = "log"), data = data_mix)* **AIC: 338.61**				
(Intercept)	2.141	0.032	67.036	<0.001 ***
Position	-0.054	0.006	-9.718	<0.001 ***
Dec	-0.079	0.034	-2.311	0.023 *
Feb	-0.198	0.036	-5.440	<0.001 ***
Jun	-0.085	0.034	-2.499	0.014 *
Null deviance: 203.055 on 127 df, Residual deviance: 96.138 on 123 df				
(C)*glm(formula = log_TuMV ~ Position, family = gaussian(link = "log"), data = data_mix)* **AIC: 360.9**				
(Intercept)	2.060	0.028	73.564	<0.001 ***
Position	-0.056	0.006	-9.074	<0.001 ***
Null deviance: 203.05 on 127 df, Residual deviance: 119.92 on 126 df				

*** p <0.001
** p < 0.01
* p < 0.05

Supplementary Figure S4. The results of generalised linear model (GLM) applied for four-time seasonal measurements of TuMV distribution within plants. (**a**) The virus accumulation at different leaf positions for plants collected in Dec. (blue), Feb. (red), Apr. (black), and Jun.(green). Symbols and lines represent actual data and leaf position effect

estimated by the full GLM model, respectively. (**b**) The effect of leaf position was evaluated by comparing three models. The full model included effects of position, sampling month, and interaction between position and month. The model were compared those with position and month effects and with position effect only. Gaussian error distribution and log link function were assumed. The full model was selected as best one by comparing Akaike’s information criteria (AIC).

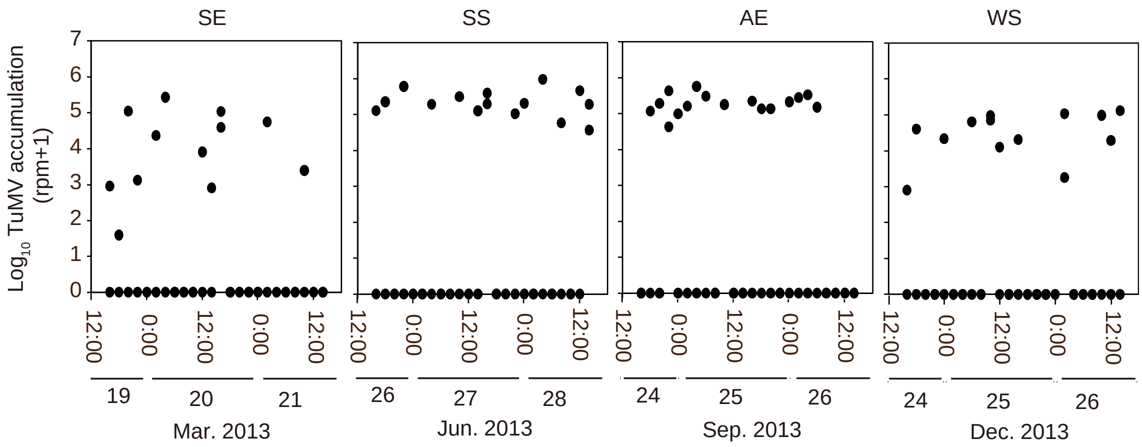

Supplementary Figure S5

TuMV accumulation in infected and uninfected plants and distribution of their sampling times during 48 h sampling periods. The 48 h samplings (starting from 16:00 on the first day) were performed at the spring equinox (SE), summer solstice (SS), autumn equinox (AE), and winter solstice (WS).

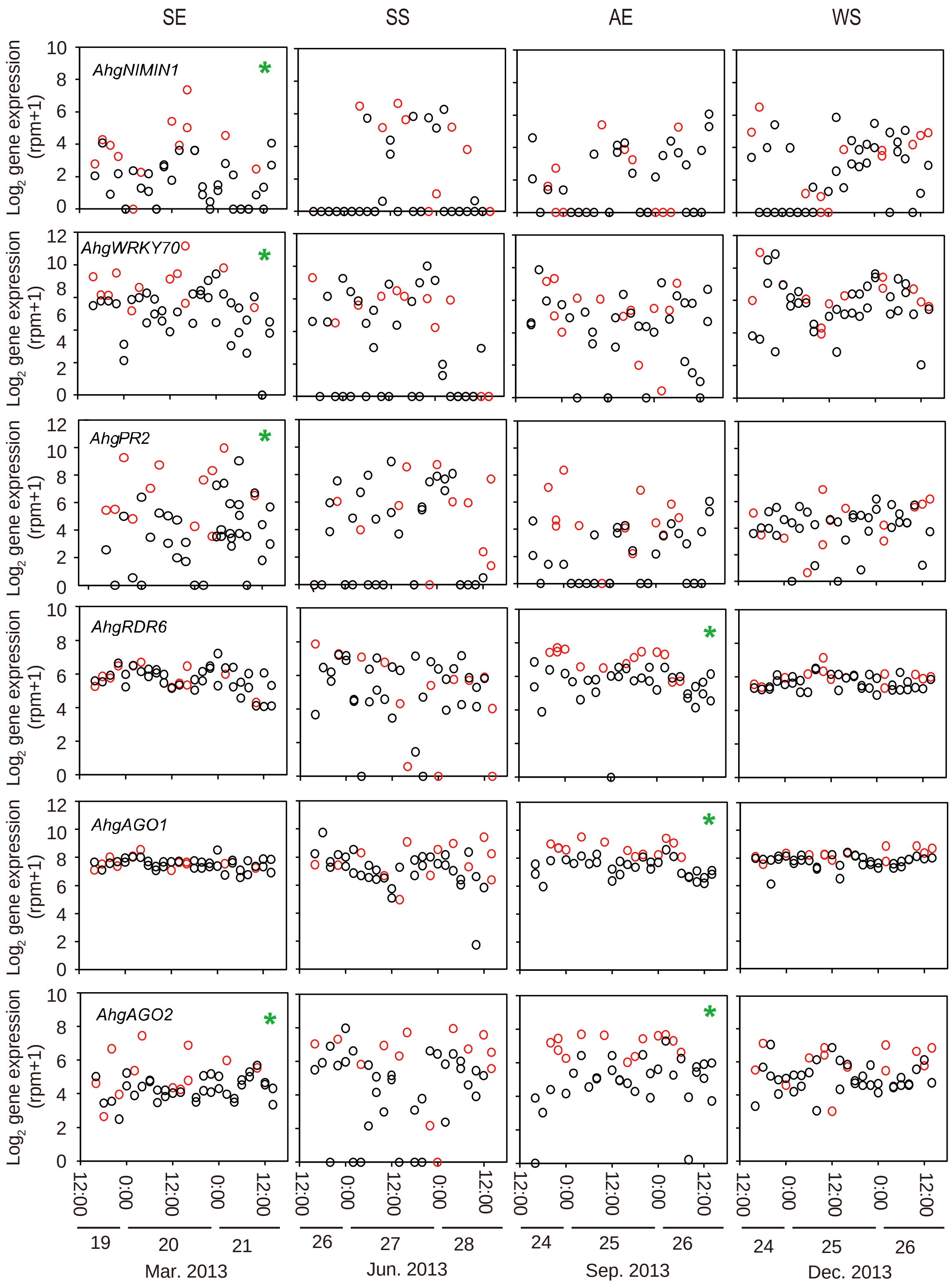

Supplementary Figure S6

Diurnal gene expression patterns of three representative SE defence DEGs (*AhgNIMIN1*, *AhgWRKY70*, and *AhgPR2*), and three representative AE defence DEGs (*AhgRDR6*,

AhgAGO1, and *AhgAGO2*) in infected (red circles) and uninfected (black circles) plants. The 48 h samplings (starting from 16:00 on the first day) were performed at the spring equinox (SE), summer solstice (SS), autumn equinox (AE), and winter solstice (WS). Significant differences in gene expressions between infected and uninfected plants were detected in the panels with a green asterisk.
