# Supplementary material for: Seasonality of interactions between a plant virus and its host during persistent infection in a natural environment
Source: ISME J. 2019 Oct 30;14(2):506–18. doi: 10.1038/s41396-019-0519-4 (PMC6976672; doi:10.1038/s41396-019-0519-4)
Supplement: Supplementary file 3 — Supplementary Tables [file 41396_2019_519_MOESM3_ESM.pdf]

Supplementary Table S1. List of samples used in the three-year time-series observation on TuMV abundance. + represents samples used in the corresponding analyses. * indicates previously published RNA-Seq data (Nagano *et al.* 2019; accession number; DRA005871, DRA005872, DRA005873), and others are newly obtained data (accession number; DRA008908) in this study.

Plant patch	Sampling date	qPCR	Strand specific qPCR (-)	RNA-seq	TuMV read-cover rate (depth $\geq$ 3, RNA-seq)	phylogenetic analysis (read-cover > 90.0)
P1	2012/9/25	+	+	+*	23.8	
P1	2012/10/4			+*	95.7	+
P1	2012/10/23	+	+	+*	63.3	
P1	2012/10/30			+*	0	
P1	2012/11/6	+		+*	41.4	
P1	2012/11/13			+*	0	
P1	2012/11/21	+	+	+*	96.9	+
P1	2012/11/27			+*	15.5	
P1	2012/12/4	+		+*	87.0	
P1	2012/12/11			+*	21.8	
P1	2012/12/18	+	+	+*	0	
P1	2012/12/25			+*	0	
P1	2013/1/8	+		+*	0	
P1	2013/1/15			+*	0	
P1	2013/1/22	+	+	+*	0	
P1	2013/1/29			+*	12.8	
P1	2013/2/5	+		+*	0	
P1	2013/2/12			+*	1.0	
P1	2013/2/19	+		+*	52.0	
P1	2013/2/26		+	+*	41.3	
P1	2013/3/7	+		+*	0	
P1	2013/3/12			+*	2.0	
P1	2013/3/19	+	+	+*	53.3	
P1	2013/3/26			+*	56.0	
P1	2013/4/2	+		+*	87.1	
P1	2013/4/9			+*	77.1	
P1	2013/4/16	+	+	+*	86.6	
P1	2013/4/23			+*	93.3	+
P1	2013/4/30	+		+*	98.7	+
P1	2013/5/7			+*	99.4	+
P1	2013/5/13	+	+	+*	99.4	+
P1	2013/5/21			+*	99.3	+
P1	2013/5/28	+		+*	98.9	+
P1	2013/6/4			+*	98.8	+
P1	2013/6/11	+	+	+*	99.0	+
P1	2013/6/19			+*	99.0	+
P1	2013/6/26	+		+*	99.2	+
P1	2013/7/9	+	+			
P1	2013/7/23	+				
P1	2013/8/6	+	+			
P1	2013/8/20	+				
P1	2013/9/17	+	+			
P1	2013/10/1	+				
P1	2013/10/15	+	+	+	87.8	
P1	2013/10/29	+				
P1	2013/11/12	+	+			
P1	2013/11/26	+		+	81.9	
P1	2013/12/10	+	+			
P1	2013/12/24	+				
P1	2014/1/14	+	+			
P1	2014/1/28	+		+	65.3	
P1	2014/2/11	+	+			
P1	2014/2/25	+				
P1	2014/3/11	+	+			
P1	2014/3/25	+				
P1	2014/4/7	+	+			
P1	2014/4/22	+				
P1	2014/5/9	+	+	+	89.7	
P1	2014/5/20	+				
P1	2014/6/3	+	+			
P1	2014/6/16	+				
P1	2014/7/1	+	+	+	91.6	+
P1	2014/7/15	+				
P1	2014/7/29	+	+			
P1	2014/8/11	+				
P1	2014/8/26	+	+	+	0	
P1	2014/9/9	+				
P1	2014/9/22	+	+	+	97.2	+
P1	2014/10/7	+				
P1	2014/10/21	+	+	+	96.9	+
P1	2014/10/28	+				

P1	2014/11/11	+				
P1	2014/11/25	+	+			
P1	2014/12/9	+				
P1	2014/12/22	+	+			
P1	2015/1/6	+				
P1	2015/1/20	+	+			
P1	2015/2/3	+		+	66.4	
P1	2015/2/17	+	+			
P1	2015/3/3	+				
P1	2015/3/17	+	+	+	0	
P1	2015/4/1	+				
P1	2015/4/14	+	+			
P1	2015/4/28	+				
P1	2015/5/12	+	+	+	92.4	+
P1	2015/5/26	+	+			
P1	2015/6/9	+				
P1	2015/6/23	+				
P1	2015/7/7	+				
P1	2015/7/21	+				
P1	2015/8/4	+				
P1	2015/8/18	+	+	+	99.1	+
P1	2015/9/1	+				
P1	2015/9/15	+	+			
P1	2015/9/29	+				
P2	2012/9/25	+	+	+*	98.1	+
P2	2012/10/4			+*	33.2	
P2	2012/10/23	+	+	+*	65.9	
P2	2012/10/30			+*	42.1	
P2	2012/11/6	+		+*	96.2	+
P2	2012/11/13			+*	69.4	
P2	2012/11/21	+	+	+*	0	
P2	2012/11/27			+*	0	
P2	2012/12/4	+		+*	0	
P2	2012/12/11			+*	72.6	
P2	2012/12/18	+	+	+*	19.4	
P2	2012/12/25			+*	0	
P2	2013/1/8	+		+*	0	
P2	2013/1/15			+*	1.5	
P2	2013/1/22	+	+	+*	0	
P2	2013/1/29			+*	81.9	
P2	2013/2/5	+		+*	0	
P2	2013/2/12			+*	0	
P2	2013/2/19	+		+*	0	
P2	2013/2/26		+	+*	0	
P2	2013/3/7			+*	0	
P2	2013/3/12			+*	0	
P2	2013/3/19	+	+	+*	0.5	
P2	2013/3/26			+*	11.9	
P2	2013/4/2	+		+*	95.5	+
P2	2013/4/9			+*	94.8	+
P2	2013/4/16	+	+	+*	89.0	
P2	2013/4/23			+*	96.3	+
P2	2013/4/30	+		+*	99.4	+
P2	2013/5/7			+*	99.3	+
P2	2013/5/13	+	+	+*	99.7	+
P2	2013/5/21			+*	0	
P2	2013/5/28	+		+*	98.8	+
P2	2013/6/4			+*	97.0	+
P2	2013/6/11	+	+	+*	99.4	+
P2	2013/6/19			+*	99.4	+
P2	2013/6/26	+		+*	99.4	+
P2	2013/7/9	+	+			
P2	2013/7/23	+				
P2	2013/8/6	+	+			
P2	2013/8/20	+				
P2	2013/9/17	+	+			
P2	2013/10/1	+				
P2	2013/10/15	+	+	+	98.8	+
P2	2013/10/29	+				
P2	2013/11/12	+	+			
P2	2013/11/26	+		+	89.2	
P2	2013/12/10	+	+			
P2	2013/12/24	+				
P2	2014/1/14	+	+	+	65.9	
P3	2014/4/22	+				
P3	2014/5/9	+	+	+	91.9	+
P3	2014/5/20	+				
P3	2014/6/3	+	+	+	0.0	
P3	2014/6/16	+				

P3	2014/7/1	+	+			
P3	2014/7/15	+				
P3	2014/7/29	+				
P3	2014/8/11	+	+	+	98.4	+
P3	2014/8/26	+	+			
P3	2014/9/9	+				
P3	2014/9/22	+	+	+	97.3	+
P3	2014/10/7	+				
P3	2014/10/21	+	+			
P3	2014/10/28	+				
P3	2014/11/11	+		+	97.8	+
P3	2014/11/25	+	+			
P3	2014/12/9	+				
P3	2014/12/22	+	+			
P3	2015/1/6	+		+	47.0	
P3	2015/1/20	+	+			
P3	2015/2/3	+				
P3	2015/2/17	+	+			
P3	2015/3/3	+				
P3	2015/3/17	+	+			
P3	2015/4/1	+				
P3	2015/4/14	+	+	+	98.7	+
P3	2015/4/28	+				
P3	2015/5/12	+	+	+	0	
P3	2015/5/26	+				
P3	2015/6/9	+				
P3	2015/6/23	+	+			
P3	2015/7/7	+				
P3	2015/7/21	+	+			
P3	2015/8/4	+				
P3	2015/8/18	+				
P3	2015/9/1	+	+			
P3	2015/9/15	+	+			
P3	2015/9/29	+		+	90.5	+

Supplementary Table S2. List of samples used in field observation of TuMV amounts in leaves at different positions within plants.

Sampling date	Plant ID	Plant stage	Leaf numbers
2015/12/22	1	Rosette	11
2015/12/22	2	Rosette	17
2015/12/22	3	Rosette	17
2015/12/22	4	Rosette	12
2016/2/23	5	Rosette	11
2016/2/23	6	Rosette	17
2016/2/23	7	Rosette	10
2016/2/23	8	Rosette	11
2016/4/19	9	Flowering	14
2016/4/19	10	Flowering	12
2016/4/19	11	Flowering	8
2016/4/19	12	Flowering	14
2016/6/21	13	Newly-formed rosette	9
2016/6/21	14	Newly-formed rosette	9
2016/6/21	15	Newly-formed rosette	10
2016/6/21	16	Newly-formed rosette	10

Supplementary Table S3. Haplotype diversity, nucleotide polymorphism (θ) and nucleotide diversity (π) of TuMV sequences calculated for among and within pant patches.

	Among plant patches (Within population)	Within plant patches		
		P1	P2	P3
Haplotype diversity (Hd)	1.00	0.60	0.79	1.00
Nucleotide polymorphism (θ)	2.9×10^{-2} ($\pm 1.2 \times 10^{-2}$)	3.0×10^{-4} ($\pm 1.5 \times 10^{-4}$)	2.6×10^{-4} ($\pm 1.3 \times 10^{-4}$)	5.8×10^{-4} ($\pm 3.1 \times 10^{-4}$)
Nucleotide diversity (π)	2.4×10^{-2} ($\pm 0.3 \times 10^{-2}$)	1.2×10^{-4} ($\pm 0.4 \times 10^{-4}$)	1.9×10^{-4} ($\pm 0.4 \times 10^{-4}$)	5.3×10^{-4} ($\pm 1.0 \times 10^{-4}$)

Sample numbers, which were used for data analysis, were 10, 17, 14, and 6 for among patches, P1, P2, and P3, respectively. Total number of sites (bp) excluding gaps / missing data were 8,205, 7,760, 8,629, and 8,303 for among patches, P1, P2, and P3, respectively. Standard deviations are in parentheses.

Supplementary Table S4. List of DEGs between TuMV-infected and uninfected plants on spring and autumn equinoxes (SE and AE) in the field seasonal transcriptome. DGEs with adjusted *P* < 0.05 are listed in the ascending order of *P* value. There was no DEG on summer and winter solstices (SS and WS).

Gene ID (Nagano *et al*., 2019)	AGI code for *Arabidopsis thaliana* orthologue	Short description of the orthologue in *A. thaliana*	Gene alias of the orthologue in *A. thaliana*	LogFC	Ajusted *P* value	Season
Ahg943027	AT4G11650.1	osmotin 34	*ATOSM34,OSM34*	2.26	5.E-07	SE
Ahg496228	AT5G60800.1	Heavy metal transport/detoxification superfamily protein		1.95	1.E-05	SE
Ahg470205	AT1G02450.1	NIM1-interacting 1	*NIMIN-1,NIMIN1*	2.77	9.E-03	SE
Ahg471490	AT1G13340.1	Regulator of Vps4 activity in the MVB pathway protein	*IST1-LIKE 6, ISTL6*	3.97	2.E-02	SE
Ahg356293	-			4.46	2.E-02	SE
Ahg473645	AT1G31280.1	Argonaute family protein	*AGO2*	1.12	2.E-02	SE
Ahg484149	AT2G02230.1	phloem protein 2-B1	*AtPP2-B1,PP2-B1*	1.35	2.E-02	SE
Ahg486080	AT3G56400.1	WRKY DNA-binding protein 70	*ATWRKY70,WRKY70*	1.61	2.E-02	SE
Ahg486168	AT3G57260.1	beta-1,3-glucanase 2	*AtPR2,BG2,BGL2,PR-2,PR2*	2.30	2.E-02	SE
Ahg488150	AT5G13320.1	Auxin-responsive GH3 family protein	*GDG1,GH3.12,PBS3,WIN3*	1.33	2.E-02	SE
Ahg495345	AT5G52810.1	NAD(P)-binding Rossmann-fold superfamily protein		1.14	2.E-02	SE
Ahg940548	AT5G10760.1	Eukaryotic aspartyl protease family protein		1.85	2.E-02	SE
Ahg350885	AT5G17760.2	P-loop containing nucleoside triphosphate hydrolases superfamily protein		1.15	5.E-02	SE
Ahg486111	AT3G56710.1	sigma factor binding protein 1	*SIB1*	1.37	5.E-02	SE
Ahg473977	AT1G48410.3	Stabilizer of iron transporter SufD / Polynucleotidyl transferase	*AGO1*	1.10	2.E-04	AE
Ahg473645	AT1G31280.1	Argonaute family protein	*AGO2*	1.53	5.E-04	AE
Ahg908972	AT1G73602.1	conserved peptide upstream open reading frame 32	*CPuORF32*	-2.92	5.E-04	AE
Ahg484149	AT2G02230.1	phloem protein 2-B1	*AtPP2-B1,PP2-B1*	1.80	4.E-03	AE
Ahg894395	AT1G52030.1	myrosinase-binding protein 2	*F-ATMBP,MBP1.2,MBP2*	5.48	4.E-03	AE
Ahg917381	AT5G56870.1	beta-galactosidase 4	*BGAL4*	2.89	4.E-03	AE
Ahg933821	AT2G32530.1	cellulose synthase-like B3	*ATCSLB03,ATCSLB3,CSLB03*	2.08	4.E-03	AE
Ahg477829	AT3G05400.1	Major facilitator superfamily protein		-2.02	4.E-03	AE
Ahg474012	AT1G48760.2	delta-adaptin	*delta-ADR,PAT4*	-1.41	5.E-03	AE
Ahg484384	AT3G26170.1	cytochrome P450, family 71, subfamily B, polypeptide 19	*CYP71B19*	3.84	5.E-03	AE
Ahg489420	AT5G25610.1	BURP domain-containing protein	*ATRD22,RD22*	-1.08	5.E-03	AE
Ahg939077	AT3G60420.1	Phosphoglycerate mutase family protein		2.51	5.E-03	AE
Ahg943255	AT4G04840.1	methionine sulfoxide reductase B6	*ATMSRB6,MSRB6*	-3.25	5.E-03	AE
Ahg479067	AT3G16130.1	RHO guanyl-nucleotide exchange factor 13	*ATROPGEF13,PIRF2,ROPGEF13*	-1.01	6.E-03	AE
Ahg918881	AT1G02305.1	Cysteine proteinases superfamily protein		1.59	6.E-03	AE
Ahg328029	AT4G37400.1	cytochrome P450, family 81, subfamily F, polypeptide 3	*CYP81F3*	-2.13	7.E-03	AE
Ahg480887	AT2G20570.1	GBF's pro-rich region-interacting factor 1	*ATGLK1,GLK1,GPRI1*	-1.16	7.E-03	AE
Ahg892702	AT5G07700.1	myb domain protein 76	*AtMYB76,MYB76*	-4.40	7.E-03	AE
Ahg919306	AT1G06000.1	UDP-Glycosyltransferase superfamily protein		-2.17	7.E-03	AE
Ahg925873	AT5G25610.1	BURP domain-containing protein	*ATRD22,RD22*	-1.13	7.E-03	AE
Ahg946406	AT4G18010.1	myo-inositol polyphosphate 5-phosphatase 2	*5PTASE2,AT5PTASE2,IP5PII*	1.91	7.E-03	AE
Ahg949787	AT5G52310.1	low-temperature-responsive protein 78 (LTI78) / desiccation-responsive protein 29A (RD29A)	*COR78,LTI140,LTI78,RD29A*	-2.61	7.E-03	AE
Ahg950218	AT5G55620.1			-2.65	7.E-03	AE
Ahg323531	AT3G49500.1	RNA-dependent RNA polymerase 6	*RDR6,SDE1,SGS2*	1.12	8.E-03	AE
Ahg478716	AT3G13330.1	proteasome activating protein 200	*PA200*	1.07	8.E-03	AE
Ahg478884	AT3G14720.1	MAP kinase 19	*ATMPK19,MPK19*	-1.76	8.E-03	AE
Ahg490817	AT4G37980.1	elicitor-activated gene 3-1	*ATCAD7,CAD7,ELI3,ELI3-1*	-1.71	8.E-03	AE
Ahg945300	AT4G27520.1	early nodulin-like protein 2	*AtENODL2,ENODL2*	-1.75	8.E-03	AE
Ahg471022	AT1G09310.1	Protein of unknown function, DUF538		-0.92	9.E-03	AE
Ahg487251	AT5G04720.1	ADR1-like 2	*ADR1-L2,PHX21*	1.04	9.E-03	AE
Ahg929203	AT3G14230.2	related to AP2 2	*RAP2.2*	1.01	9.E-03	AE
Ahg488339	AT5G14950.1	golgi alpha-mannosidase II	*ATGMII,GMII*	0.92	9.E-03	AE
Ahg471997	AT1G17840.1	white-brown complex homolog protein 11	*ABCG11,AtABCG11,ATWBC11,COF1,DSO,*[illegible]	-0.90	1.E-02	AE
Ahg485933	AT3G55120.1	Chalcone-flavanone isomerase family protein	*A11,CFI,TT5,CHI*	-3.23	1.E-02	AE
Ahg494762	AT5G42720.1	Glycosyl hydrolase family 17 protein		-1.39	1.E-02	AE
Ahg496967	AT5G66400.1	Dehydrin family protein	*ATDI8,RAB18*	-3.12	1.E-02	AE
Ahg933965	AT2G33770.1	phosphate 2	*ATUBC24,PHO2,UBC24*	1.26	1.E-02	AE
Ahg897209	AT4G33790.1	Jojoba acyl CoA reductase-related male sterility protein	*CER4,FAR3,G7*	-2.50	1.E-02	AE
Ahg473464	AT1G32780.1	GroES-like zinc-binding dehydrogenase family protein		-6.67	1.E-02	AE
Ahg934322	AT2G36880.1	methionine adenosyltransferase 3	*MAT3*	-1.17	1.E-02	AE
Ahg491707	AT4G30450.1	glycine-rich protein		-2.22	1.E-02	AE
Ahg901436	AT2G35860.1	FASCICLIN-like arabinogalactan protein 16 precursor	*FLA16*	-2.24	1.E-02	AE
Ahg485670	AT3G52960.1	Thioredoxin superfamily protein		-1.25	1.E-02	AE
Ahg485476	AT3G51240.1	flavanone 3-hydroxylase	*F3'H,F3H,TT6*	-2.78	1.E-02	AE
Ahg937924	AT3G50760.1	galacturonosyltransferase-like 2	*GATL2*	-4.55	1.E-02	AE
Ahg489007	AT5G20960.2	aldehyde oxidase 1	*AAO1,AO1,AOalpha,AT-AO1,ATAO,AtAO1*	1.20	1.E-02	AE
Ahg482978	AT2G39570.1	ACT domain-containing protein	*ACR9*	1.16	2.E-02	AE
Ahg482040	AT2G31110.2	Plant protein of unknown function (DUF828)	*TBL40*	2.44	2.E-02	AE
Ahg925482	AT3G20362.1			-1.58	2.E-02	AE
Ahg939363	AT3G62740.1	beta glucosidase 7	*BGLU7*	-2.03	2.E-02	AE
Ahg943195	AT4G04020.1	fibrillin	*FIB*	-2.51	2.E-02	AE
Ahg482632	AT2G36540.1	Haloacid dehalogenase-like hydrolase (HAD) superfamily protein		1.38	2.E-02	AE
Ahg935883	AT2G01620.1	RNI-like superfamily protein	*MEE11*	0.91	2.E-02	AE
Ahg471490	AT1G13340.1	Regulator of Vps4 activity in the MVB pathway protein	*IST1-LIKE 6, ISTL6*	3.48	2.E-02	AE
Ahg485388	AT3G50370.1			0.65	2.E-02	AE
Ahg930439	AT3G23820.1	UDP-D-glucuronate 4-epimerase 6	*GAE6*	-0.85	2.E-02	AE
Ahg481994	AT2G30770.1	cytochrome P450, family 71, subfamily A, polypeptide 13	*CYP71A13*	3.14	2.E-02	AE
Ahg945904	AT4G22240.1	Plastid-lipid associated protein PAP / fibrillin family protein		1.29	2.E-02	AE
Ahg941951	AT5G24160.1	squalene monoxygenase 6	*SQE6*	-1.53	2.E-02	AE
Ahg939809	AT5G04120.1	Phosphoglycerate mutase family protein		-3.97	2.E-02	AE
Ahg929130	AT3G13750.1	beta galactosidase 1	*BGAL1,BGAL1*	1.39	2.E-02	AE
Ahg942295	AT5G27920.1	F-box family protein		2.87	2.E-02	AE
Ahg944988	AT4G30080.1	auxin response factor 16	*ARF16*	1.16	2.E-02	AE
Ahg495611	AT5G55250.1	IAA carboxylmethyltransferase 1	*AtIAMT1,IAMT1*	-3.60	2.E-02	AE
Ahg927205	AT1G78000.2	sulfate transporter 1;2	*SEL1,SULTR1;2*	1.82	2.E-02	AE
Ahg477021	AT1G78570.1	rhamnose biosynthesis 1	*ATRHM1,RHM1,ROL1*	-1.52	3.E-02	AE
Ahg935524	AT2G45660.1	AGAMOUS-like 20	*AGL20,ATSOC1,SOC1*	1.51	3.E-02	AE
Ahg914721	AT1G55910.1	zinc transporter 11 precursor	*ZIP11*	-1.04	3.E-02	AE
Ahg346572	AT2G47730.1	glutathione S-transferase phi 8	*ATGSTF5,ATGSTF8,GST6,GSTF8*	-1.01	3.E-02	AE
Ahg356368	AT5G46330.1	Leucine-rich receptor-like protein kinase family protein	*FLS2*	2.19	3.E-02	AE
Ahg481471	AT2G26150.1	heat shock transcription factor A2	*ATHSFA2,HSFA2*	-6.15	3.E-02	AE
Ahg487705	AT5G08640.1	flavonol synthase 1	*ATFLS1,FLS,FLS1*	-2.27	3.E-02	AE
Ahg490638	AT4G38740.1	rotamase CYP 1	*ROC1*	-1.27	3.E-02	AE
Ahg891467	AT5G41900.1	alpha/beta-Hydrolases superfamily protein		-1.31	3.E-02	AE
Ahg930777	AT2G04570.1	GDSL-like Lipase/Acylhydrolase superfamily protein		-1.73	3.E-02	AE
Ahg946531	AT4G17030.1	expansin-like B1	*AT-EXPR,ATEXLB1,ATEXPR1,ATHEXP BE*[illegible]	-2.37	3.E-02	AE
Ahg899338	AT3G63440.1	cytokinin oxidase/dehydrogenase 6	*ATCKX6,ATCKX7,CKX6*	-2.38	3.E-02	AE
Ahg474340	AT1G52230.1	photosystem I subunit H2	*PSAH-2,PSAH2,PSI-H*	-0.75	4.E-02	AE
Ahg938584	AT3G56290.1			-3.51	4.E-02	AE
Ahg488652	AT5G17860.1	calcium exchanger 7	*CAX7*	1.96	4.E-02	AE
Ahg937908	AT3G50685.1			-0.83	4.E-02	AE
Ahg948884	AT5G43150.1			-2.08	4.E-02	AE
Ahg478305	AT3G09820.1	adenosine kinase 1	*ADK1,ATADK1*	-1.07	4.E-02	AE
Ahg488016	AT5G11790.1	N-MYC downregulated-like 2	*NDL2*	-1.43	4.E-02	AE
Ahg490836	AT4G37800.1	xyloglucan endotransglucosylase/hydrolase 7	*XTH7*	-1.56	4.E-02	AE
Ahg947030	AT4G13930.1	serine hydroxymethyltransferase 4	*SHM4*	-0.94	4.E-02	AE
Ahg475149	AT1G61800.1	glucose-6-phosphate/phosphate translocator 2	*ATGPT2,GPT2*	-4.75	4.E-02	AE
Ahg486810	AT3G63060.1	EID1-like 3	*EDL3*	-4.46	4.E-02	AE
Ahg949485	AT5G49740.1	ferric reduction oxidase 7	*ATFRO7,FRO7*	0.86	4.E-02	AE
Ahg950456	AT5G57655.2	xylose isomerase family protein		0.92	4.E-02	AE

Ahg490167	AT4G05180.1	photosystem II subunit Q-2	*PSBQ,PSBQ-2,PSII-Q*	-0.70	5.E-02	AE
Ahg482690	AT2G37040.1	PHE ammonia lyase 1	*ATPAL1,PAL1*	-1.63	5.E-02	AE
Ahg316086	AT1G71000.1	Chaperone DnaJ-domain superfamily protein		-6.33	5.E-02	AE
Ahg326101	AT4G22100.1	beta glucosidase 2	*BGLU3*	-2.08	5.E-02	AE
Ahg326139	AT5G17050.1	UDP-glucosyl transferase 78D2	*UGT78D2*	-1.73	5.E-02	AE
Ahg341522	AT3G13900.1	ATPase E1-E2 type family protein / haloacid dehalogenase-like hydrolase family protein		2.28	5.E-02	AE
Ahg475766	AT1G66700.1	S-adenosyl-L-methionine-dependent methyltransferases superfamily protein	*PXMT1*	2.78	5.E-02	AE
Ahg479246	AT3G17650.1	YELLOW STRIPE like 5	*PDE321,YSL5*	0.72	5.E-02	AE
Ahg483684	AT2G45470.1	FASCICLIN-like arabinogalactan protein 8	*AGP8,FLA8*	-1.19	5.E-02	AE
Ahg484456	AT3G26810.1	auxin signaling F-box 2	*AFB2*	0.81	5.E-02	AE
Ahg491000	AT4G36360.1	beta-galactosidase 3	*BGAL3*	-1.26	5.E-02	AE
Ahg937262	-			3.38	5.E-02	AE
Ahg941077	AT5G15870.1	glycosyl hydrolase family 81 protein		1.09	5.E-02	AE

Supplementary Table S5. GO (gene ontology) analyses for SE and AE DEGs between TuMV-infected and -uninfected plants in the field seasonal transcriptome. Enriched GOs for up-regulated DEGs in SE and down-regulated DEGs in AE are listed. There were no down-regulated DEGs in SE, and no enriched GO for up-regulated DEGs in AE. Adjusted *P* < 0.01.

Season	ID	Description	Number of DEG[a]	A[b]	B[c]	U[d]	Adjusted *P*
SE	GO:0042742	Defence response to bacterium	4	13	317	26927	3.7.E-02
	GO:0009816	Defence response to bacterium, incompatible interaction	3	13	54	26927	1.4.E-02
	GO:0010112	Regulation of systemic acquired resistance	2	13	13	26927	3.7.E-02
AE	GO:0080167	Response to karrikin	6	62	143	26927	6.5.E-03
	GO:0009813	Favonoid biosynthetic process	4	62	41	26927	8.1.E-03
	GO:0055114	Oxidation-reduction process	12	62	1264	26927	4.5.E-02
	GO:0009269	Response to desiccation	3	62	28	26927	4.9.E-02
	GO:0045486	Naringenin 3-dioxygenase activity	2	62	2	26927	1.2.E-02

a: DEGs in the corresponding GO

b: Total numbers of up-regulated DEGs in SE and down-regulated DEGs in AE

c: Genes in the corresponding GO

d: Total number of annotated genes
